# Direct endoscopic necrosectomy with the newly developed 6-mm powered rotating resection catheter: When size matters

**DOI:** 10.1055/a-1968-6966

**Published:** 2022-11-25

**Authors:** Gianenrico Rizzatti, Mario Gagliardi, Giulia Tripodi, Michele Impagnatiello, Antonio Gasbarrini, Guido Costamagna, Alberto Larghi

**Affiliations:** 1 Digestive Endoscopy Unit, Fondazione Policlinico Universitario A. Gemelli IRCCS, Rome, Italy; 2 Digestive Endoscopy Unit, San Giovanni di Dio e Ruggi d’Aragona University Hospital, Salerno, Italy; 3 UOC di Medicina Interna e Gastroenterologia, Dipartimento di Scienze Mediche e Chirurgiche, Fondazione Policlinico Universitario A. Gemelli IRCCS; 4 Dipartimento di Medicina e Chirurgia Traslazionale, Università Cattolica Del Sacro Cuore

A 41-year-old man with acute pancreatitis of unknown etiology developed a 17 × 8-cm walled-off necrosis (WON) that was drained percutaneously. The purulent fluid grew *Klebsiella* and *Candida albicans* . After transfer to our hospital, endoscopic ultrasound (EUS)-guided drainage was performed, and a 20 × 10-mm Axios stent was placed. The solid component of the WON was about 90 % and direct endoscopic necrosectomy (DEN) was scheduled. DEN was started with a 3.2-mm EndoRotor catheter that broke after a few minutes of use. The following day, the new 6-mm EndoRotor catheter was utilized in association with an Olympus GIF-XTQ160 scope. The catheter was placed through the Axios stent ( Fig. 1 ), and in a 70-minute procedure, it was able to aspirate all necrotic content, amounting to 800 mL of collected material ( Video 1 ).

**Fig. 1:**
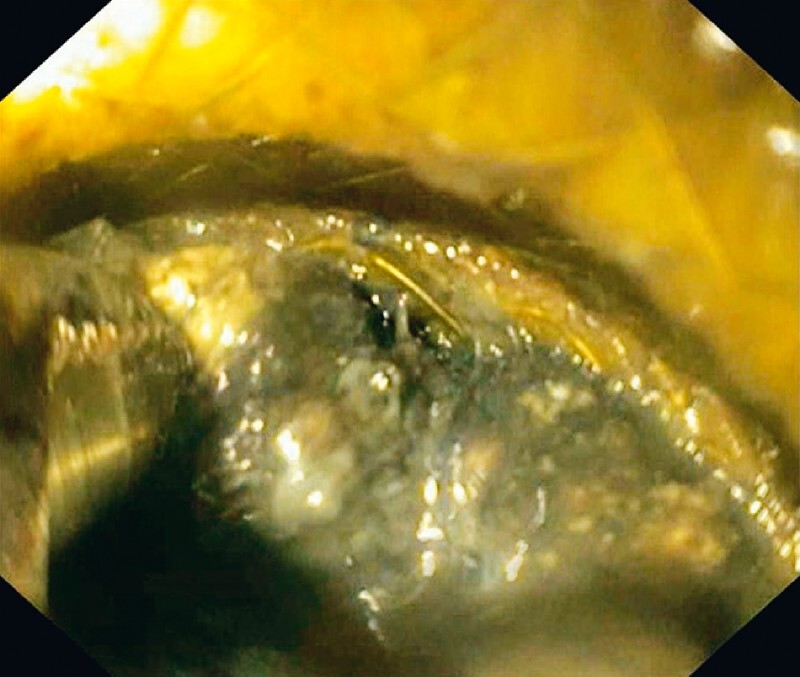
The newly developed 6-mm EndoRotor catheter placed through a lumen-apposing metal stent, to perform direct endoscopic necrosectomy in a walled-off necrosis in a patient with acute pancreatitis.

**Video 1** Direct endoscopic necrosectomy performed with the newly developed 6-mm EndoRotor catheter.


A 77-year-old woman developed biliary acute pancreatitis, complicated by the formation of a large infected WON (16 × 10 mm, necrotic content 80 %), which was drained emergently using a 20 × 10-mm Axios stent. DEN was performed 3 days later using the 6-mm EndoRotor catheter, and in a 90-minute procedure, 90 % clearance of the necrotic content amounting to 600 mL of collected material had been achieved.

We report, for the first time, utilization of the new 6 mm EndoRotor catheter, which represents an evolved version of the 3.2-mm tool, the first dedicated device for DEN 1 2 3 . This new catheter can be used with the Olympus GIF-XTQ160 scope ( Fig. 2 a ) or with an accessory catheter channel that can be attached to an Olympus GIF290 or equivalent Fuji/Pentax scopes ( Fig. 2 b ). Compared to the 3.2-mm catheter, the 6.0-mm catheter has a 4.4-times larger cutting window and a 2.5-times larger inner lumen, which allows for an 8-times greater throughput and possibly faster and more effective DEN. The average number of procedures required to treat WON with the 3.2-mm catheter has been reported to be 2.1; this number might be decreased by use of the 6-mm device 4 .

**Fig. 2:**
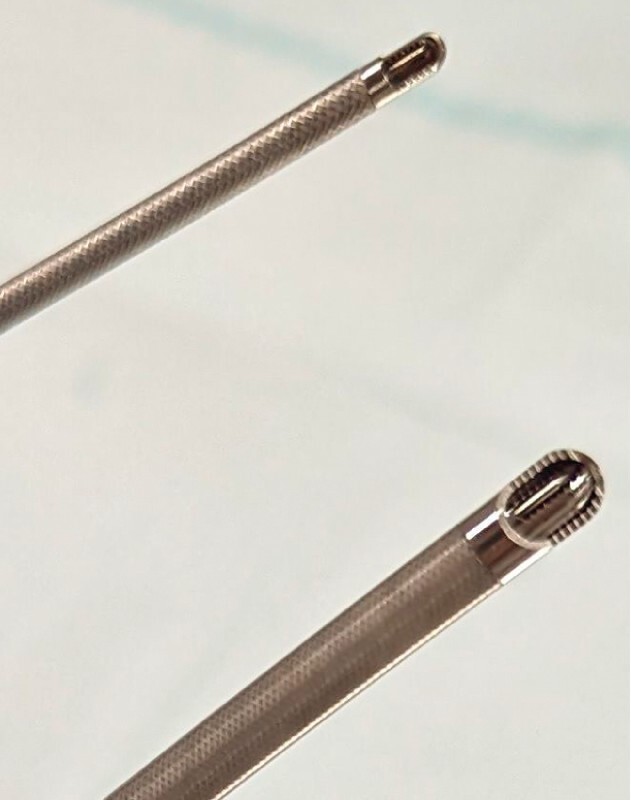
Comparison between the new 6.0 mm EndoRotor and standard 3.2 mm EndoRotor catheters.

Endoscopy_UCTN_Code_TTT_1AS_2AD

## Notes

Endoscopy E-Videos

https://eref.thieme.de/e-videos
*Endoscopy E-Videos* is an open access online section, reporting on interesting cases and new techniques in gastroenterological endoscopy. All papers include a high quality video and all contributions are freely accessible online. Processing charges apply (currently EUR 375), discounts and wavers acc. to HINARI are available. This section has its own submission website at https://mc.manuscriptcentral.com/e-videos

## References

[1] van der Wiel S E Poley J W Grubben M JAL The EndoRotor, a novel tool for the endoscopic management of pancreatic necrosis Endoscopy 2018 50 E240 E241 29920619 10.1055/a-0628-6136

[2] van der Wiel S E May A Poley J W Preliminary report on the safety and utility of a novel automated mechanical endoscopic tissue resection tool for endoscopic necrosectomy: a case series Endosc Int Open 2020 8 E274 E280 32118101 10.1055/a-1079-5015 PMC7035027

[3] Rizzatti G Rimbas M Impagnatiello M Endorotor-based endoscopic necrosectomy as a rescue or primary treatment of complicated walled-off pancreatic necrosis. A case series J Gastrointestin Liver Dis 2020 29 681 684 33118541 10.15403/jgld-2534

[4] Stassen P MC de Jonge P JF Bruno M J Safety and efficacy of a novel resection system for direct endoscopic necrosectomy of walled-off pancreas necrosis: a prospective, international, multicenter trial Gastrointest Endosc 2022 95 471 479 34562471 10.1016/j.gie.2021.09.025

